# Neuro-immune-vascular interactions in rosacea and hypertension: a mechanistic review

**DOI:** 10.3389/fimmu.2026.1883039

**Published:** 2026-08-03

**Authors:** Yujiang Xie, Mei Cai

**Affiliations:** Department of Dermatology and Venereology, The Affiliated Hospital of Yunnan University, Kunming Medical University, Kunming, China

**Keywords:** hypertension, neuro-immune-vascular mechanisms, neuroinflammation, rosacea, therapeutics

## Abstract

Rosacea is a chronic inflammatory skin disorder characterized by recurrent flare-ups and significant variability in therapeutic responses. Its pathophysiology is complex and involves multilevel processes, including neurogenic dysregulation, immune activation, and alterations in vascular reactivity. Recent epidemiological studies suggest that rosacea may be associated with an increased risk of hypertension and other cardiometabolic diseases. However, it remains unclear whether this association merely reflects a comorbidity or indicates the presence of shared underlying pathophysiological mechanisms. Therefore, from an integrated neuro-immune-vascular perspective, this review summarizes current evidence regarding potential shared neuro-immune-vascular mechanisms that may be associated with rosacea and hypertension. Emerging evidence suggests that aberrant activation of sensory nerves, neuropeptide release, and subsequent innate and adaptive immune responses may collectively participate in a putative regulatory network. Locally, this network may be associated with neurogenic inflammation and aberrant vasodilation in the skin; at the systemic level, it may potentially be associated with dysregulated blood pressure control through mechanisms such as chronic low-grade inflammation, endothelial dysfunction, and vascular remodeling. By synthesizing evidence from dermatology, immunology, and vascular biology, this review summarizes potential neuro-immune-vascular interactions associated with rosacea and hypertension and discusses their possible implications for comorbidity management.

## 1 Introduction

Rosacea is a chronic inflammatory skin disorder that primarily affects the central face, including the cheeks, nose, forehead, and periorbital region. Its global prevalence is approximately 5.5%, with a higher incidence among middle-aged women. Clinically, rosacea exhibits marked heterogeneity, presenting with a spectrum of manifestations, including paroxysmal flushing, persistent erythema, telangiectasia, and papules or pustules, as well as neuropathic symptoms such as stinging, burning, and dryness (1). Beyond cutaneous manifestations, patients often experience significant social anxiety and psychological distress. Previous studies have demonstrated a high comorbidity rate of rosacea with anxiety and depression, which severely impairs patients’ physical and mental health as well as their quality of life (2). Although clinically, based on phenotypes, rosacea can be stratified into erythematotelangiectatic rosacea (ETR), papulopustular rosacea (PPR), phymatous rosacea (PHR), and ocular rosacea (OR) types for stratified management (1), the disease follows a protracted and relapsing course with marked interindividual variability in treatment responses. Current therapeutic strategies primarily target inflammation or vasodilation; nevertheless, some patients exhibit poor efficacy or rapid relapse upon treatment withdrawal, suggesting that the underlying pathogenesis remains incompletely understood (3). Recent studies suggest that rosacea may not be merely a cutaneous inflammatory disorder, but rather a multi-level pathological process involving neuroregulatory abnormalities, immune activation, and vascular dysfunction. Sensory neural hyperreactivity, increased neuropeptide release, and dysregulated vasomotor responses are considered to play important roles in disease initiation and symptom fluctuation (4). Meanwhile, systemic comorbidities associated with rosacea have garnered increasing attention. Multiple epidemiological studies suggest that rosacea may be associated with an elevated risk of cardiovascular diseases, metabolic syndrome, and hypertension, which may reflect a potential link with systemic vascular dysfunction and chronic low-grade inflammation (5). However, current evidence primarily supports a statistical correlation, while the causal relationship and its underlying biological basis remain unclear. Furthermore, variations across studies in terms of study design, population characteristics, and control of confounding factors contribute to inconsistencies in the conclusions.

Current clinical guidelines and routine dermatological practice increasingly emphasize the importance of assessing systemic comorbidities in patients with rosacea. However, this emphasis is primarily focused on risk recognition and clinical screening, whereas the extent to which these associations are integrated into a unified pathophysiological framework remains limited. Therefore, re-examining the pathogenesis of rosacea and exploring shared pathways with its comorbidities may help improve integrated management, potentially enhancing therapeutic efficacy and reducing disease burden. Traditionally, rosacea and hypertension have been studied independently within their respective disciplinary frameworks. This single-disease research paradigm may be insufficient to fully explain the observed overlap between the two conditions in terms of risk factors, timing of onset, and pathological pathways. Accordingly, this review examines rosacea and hypertension from an integrated neuro-immune-vascular perspective and summarizes current evidence regarding potential shared mechanisms involving sensory nerve activation, immune-inflammatory responses, and vascular dysfunction. This review aims to provide a hypothesis-generating mechanistic perspective on the potential relationship between rosacea and hypertension and to discuss possible implications for future comorbidity management.

## 2 Evidence for the association between rosacea and hypertension

Existing epidemiological studies generally suggest a potential association between rosacea and hypertension. A systematic review and meta-analysis including 40,752 patients with rosacea reported that mean systolic blood pressure was significantly elevated in this population (standardized mean difference [SMD] = 0.293, 95% CI: 0.054-0.532), suggesting a higher prevalence of hypertension and elevated blood pressure levels compared with healthy controls (6). A large-scale case-control study from Taiwan, including 33,533 patients with rosacea and 67,106 controls, demonstrated a modestly increased risk of hypertension among patients with rosacea (odds ratio [OR] = 1.17; 95% CI = 1.12-1.21) (7). Similarly, a cross-sectional study comparing rosacea patients with controls found that both systolic and diastolic blood pressure levels were significantly higher in the rosacea group (systolic: 123.14 ± 16.94 mmHg vs. 114.36 ± 14.11 mmHg, P = 0.045; diastolic: 80 ± 10.57 mmHg vs. 74.61 ± 6.82 mmHg, P = 0.016) (8). Supporting genetic evidence has also been explored using bidirectional Mendelian randomization analysis; however, the observed effect size was extremely modest (OR = 1.0032, 95% CI: 1.0001–1.0063), suggesting a negligible genetic contribution. In addition, this analysis was primarily conducted in populations of European ancestry, which may limit its generalizability to other ethnic groups. Overall, these findings do not support a robust causal relationship and should be interpreted as exploratory genetic evidence with limited inferential strength (9). Collectively, available evidence indicates a modest association between rosacea and hypertension, with effect estimates varying across studies but generally remaining in the low-to-moderate range. This association does not appear to reflect a strong independent risk factor and is more likely to be explained by shared underlying physiological and inflammatory pathways.

Although C-reactive protein (CRP) levels have been reported to be elevated in both rosacea and cardiovascular diseases (10, 11), CRP is a non-specific systemic inflammatory biomarker and does not reflect disease-specific pathogenic mechanisms. Therefore, its elevation should not be interpreted as direct evidence supporting shared causative pathways between rosacea and hypertension, but rather as an indicator of systemic low-grade inflammation that may be present in both conditions. Furthermore, several factors known to exacerbate rosacea symptoms, such as dietary patterns, smoking, alcohol consumption, and psychological stress, have also been identified as common risk factors for hypertension (12, 13). These observations suggest that shared lifestyle and environmental factors may act as important confounders in studies examining the association between rosacea and hypertension.

## 3 Interpreting the association between rosacea and hypertension

Taken together, the association between rosacea and hypertension is more appropriately interpreted as a correlation rather than a causal relationship. Current epidemiological evidence is derived primarily from cross-sectional and case-control studies, with only a limited number of cohort studies available. There remains a lack of prospective studies and mechanistic investigations that could definitively determine whether rosacea directly promotes the development of hypertension, or conversely, whether hypertension contributes to the onset of rosacea. Therefore, this association more likely reflects partially shared pathophysiological underpinnings between the two conditions in terms of neural regulation, vascular reactivity, and immune-inflammatory status, rather than a unidirectional causal chain. Based on available clinical observations and mechanistic evidence, three non-mutually exclusive interpretations may help explain this association.

### 3.1 Potential contribution of rosacea-associated neuroimmune stress to hypertension

Current evidence suggests that rosacea, characterized by neurogenic inflammation and vascular abnormalities, may be associated with broader systemic inflammatory and neurovascular alterations that could contribute to blood pressure dysregulation (6). Patients with rosacea often exhibit sensory neural hyperreactivity. Aberrant activation of transient receptor potential (TRP) channels can induce the release of neuropeptides, leading to local vasodilation and inflammatory responses (14). These neuropeptides may influence vascular tone through both local and systemic neurovascular signaling pathways (15). In rosacea lesional skin, infiltration and activation of mast cells, macrophages, and neutrophils have been observed. Experimental studies suggest that these immune cells may contribute to a pro-inflammatory microenvironment characterized by increased levels of inflammatory mediators, reactive oxygen species (ROS), and cytokines such as IL-6, TNF-α, and IL-1β (4, 16). Such inflammatory activity has been proposed to reflect a state of systemic low-grade inflammation, although direct evidence of systemic dissemination of skin-derived mediators remains limited. Chronic low-grade inflammation is recognized as a contributor to hypertension through mechanisms including endothelial dysfunction, oxidative stress, and altered vascular reactivity (17, 18). Taken together, current evidence suggests a potential link between rosacea-associated neuroimmune activation and systemic vascular dysregulation. However, this interpretation is primarily based on cross-sectional and indirect mechanistic evidence, and longitudinal studies establishing temporal or causal relationships remain lacking.

### 3.2 Potential effects of hypertension-associated vascular and neural remodeling on rosacea

An alternative interpretation suggests that hypertension, as a systemic vascular disorder, may promote the development or exacerbation of rosacea through microvascular structural and functional abnormalities. Patients with hypertension commonly exhibit microvascular remodeling, vascular endothelial dysfunction, and dysregulation of vasomotor regulation (19). The facial skin is highly vascularized and particularly sensitive to hemodynamic fluctuations. Sustained elevation in blood pressure and increased vascular reactivity may enhance the sensitivity of facial vessels to external stimuli, leading to typical rosacea symptoms such as flushing and erythema (20). Hypertension-related sympathetic nervous system hyperactivity and activation of the renin-angiotensin system (RAS) not only affect blood pressure regulation but may also promote cutaneous inflammatory responses via neuroimmune pathways (15). Angiotensin II (Ang II) has been demonstrated to enhance oxidative stress and inflammatory signaling, mechanisms that have also been observed in the pathogenesis of rosacea (21). Taken together, this interpretation may partly explain the increased prevalence of rosacea in patients with hypertension; however, it lacks direct causal evidence and does not fully account for cases occurring in individuals without hypertension.

### 3.3 Shared neuroimmune susceptibility as an integrative explanation

Compared with unidirectional causal hypotheses, emerging evidence suggests that rosacea and hypertension may share common abnormalities in neuro-immune-vascular regulation, which may manifest as distinct disease phenotypes across different organ systems. Aberrant sensory nerve activation, immune-inflammatory responses, and vascular dysfunction appear to represent interconnected processes underlying both conditions. Abnormal activation of TRP channels, dysregulated neuropeptide release, and sustained immune activation may represent shared biological processes underlying both conditions (18, 22–24). For example, the Th17/IL-17 signaling pathway has been demonstrated to play an important role in both rosacea lesions and hypertension-related vascular inflammation (25, 26). Furthermore, psychological stress, lifestyle factors, and genetic susceptibility may simultaneously affect cutaneous inflammation and vascular function via the central and peripheral nervous systems (27, 28), thereby explaining the phenomenon whereby the two diseases are temporally asynchronous yet frequently coexist in clinical settings at the individual level. Collectively, this interpretation may better integrate current epidemiological and mechanistic evidence regarding the coexistence of rosacea and hypertension. Compared with unidirectional hypotheses, it offers a more coherent explanation for the comorbidity of these two diseases and is more consistent with the current understanding of systemic regulatory imbalance in chronic inflammatory diseases.

## 4 The neuro-immune-vascular regulatory network in rosacea and hypertension

Although rosacea primarily manifests as a cutaneous disorder, whereas hypertension is a systemic cardiovascular disease, emerging evidence suggests that both conditions may share features of neurovascular hyperreactivity and dysregulated sensory processing (29, 30). These shared characteristics provide a conceptual basis for a neuro-immune-vascular regulatory network that may help contextualize their potential association.

From a neural regulatory perspective, transient receptor potential (TRP) channels in sensory nerve endings function as key sensors of thermal, mechanical, and chemical stimuli. Aberrant activation of these channels may contribute to dysregulated vascular reactivity and neurogenic signaling, thereby linking cutaneous responses with systemic vascular regulation (31). Neuropeptides act as key mediators transmitting sensory signals to vascular and immune systems, enabling bidirectional neurovascular-immune communication (32, 33). A key feature of this network is the context-dependent nature of neuropeptide signaling. Calcitonin gene-related peptide (CGRP) and other neuropeptides may exert divergent effects depending on tissue microenvironment: in cutaneous tissues, they are associated with vasodilation and inflammatory responses, whereas in the systemic circulation CGRP functions as an endogenous vasodilatory factor contributing to vascular homeostasis and compensatory regulation (34). This dual behavior highlights the complexity of neurovascular signaling across disease contexts (35). This concept is consistent with emerging neuroimmune frameworks proposed in other chronic inflammatory skin diseases, in which sensory nerve–immune–vascular interactions are considered central to disease maintenance rather than epiphenomena (36).

Immune cells, including mast cells, macrophages, neutrophils, and T cells, act as signal amplifiers within this network by converting transient neurogenic inputs into sustained inflammatory responses (37). In rosacea, immune activation is associated with persistent cutaneous inflammation and vascular reactivity (32), whereas in hypertension, immune-mediated chronic low-grade inflammation is recognized as a contributor to endothelial dysfunction and vascular remodeling (18). Thus, immune cells serve as an interface linking neural activation with vascular alterations in both conditions.

**Figure 1:**
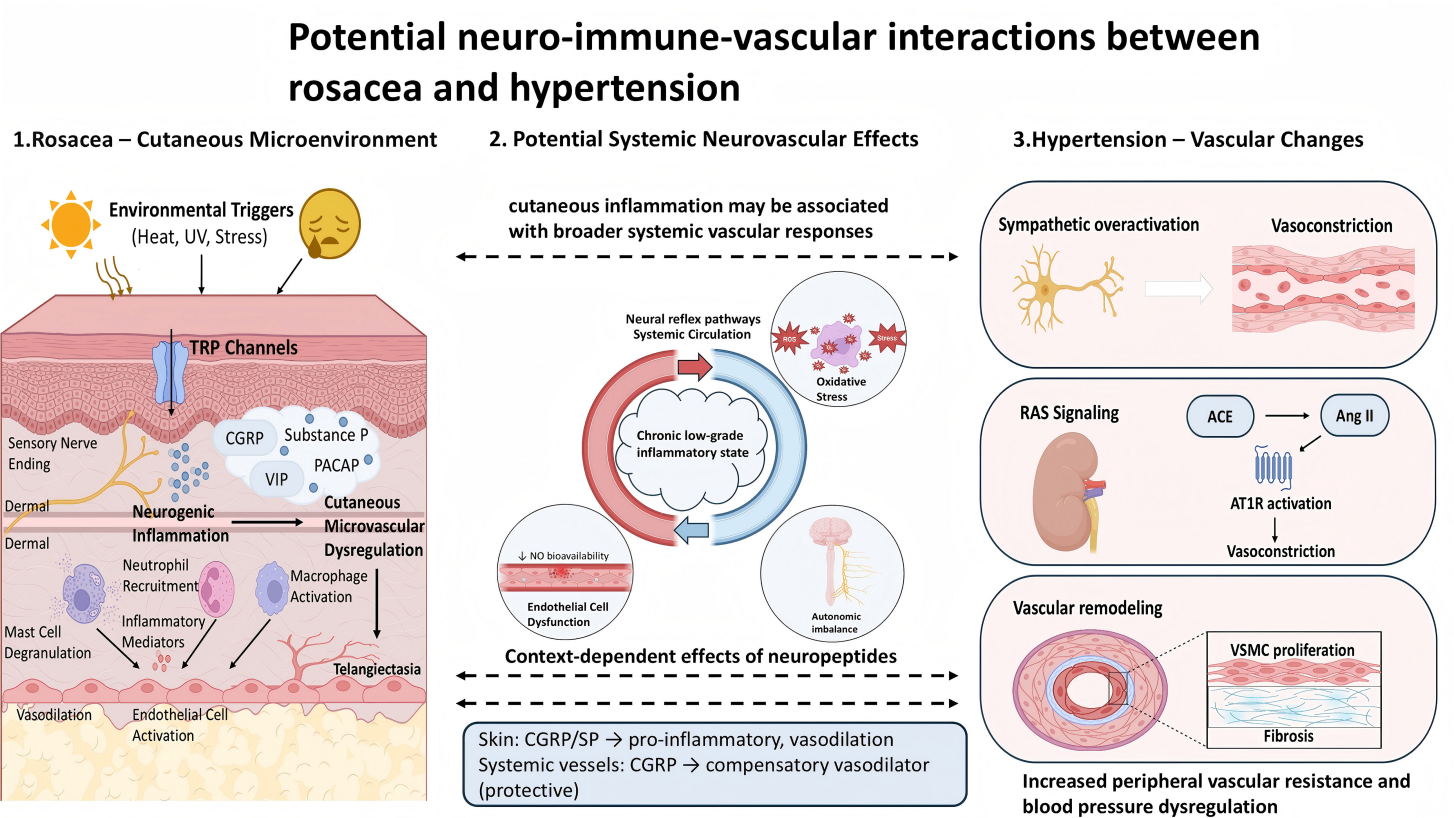
Potential neuro-immune-vascular interactions between rosacea and hypertension. This schematic summarizes potential neuro-immune-vascular processes that may be relevant to both rosacea and hypertension. In the cutaneous microenvironment of rosacea (left), environmental triggers such as heat, ultraviolet radiation, and emotional stress may activate TRP channels on sensory nerve endings, promoting the release of neuropeptides including CGRP, substance P, VIP, and PACAP. These mediators may contribute to neurogenic inflammation, endothelial activation, immune cell recruitment, vasodilation, and cutaneous microvascular dysregulation. Persistent local inflammation has been proposed to be associated with broader neurovascular and inflammatory responses through systemic circulation or neural reflex pathways (middle), potentially contributing to oxidative stress, autonomic imbalance, endothelial dysfunction, and a chronic low-grade inflammatory state. In the systemic vasculature (right), sympathetic overactivation, RAS signaling, vascular remodeling, VSMC proliferation, fibrosis, and increased peripheral vascular resistance have been implicated in blood pressure dysregulation. Neuropeptides such as CGRP may exhibit context-dependent effects, acting as pro-inflammatory mediators in cutaneous inflammation while potentially exerting compensatory vasodilatory effects in systemic vessels. Collectively, current evidence suggests that rosacea and hypertension may involve partially overlapping neuro-immune-vascular processes rather than a direct unidirectional causal relationship. This schematic is intended to be hypothesis-generating and does not imply a unified causal pathway.

Although rosacea is primarily a localized skin disease, emerging evidence suggests that neurogenic and immune activation may not be strictly confined to the skin. Inflammatory mediators and neuropeptides may influence systemic vascular function through circulating pathways or neural reflex mechanisms, contributing to a state of low-grade systemic inflammation and altered vascular homeostasis. Collectively, these observations support a conceptual framework in which rosacea and hypertension may share partially overlapping neuro-immune-vascular regulatory features rather than representing entirely independent disease entities, as illustrated in **Figure 1**.

## 5 Shared neuroimmune pathways potentially linking rosacea and hypertension

### 5.1 Interactions between neurosensory abnormalities and vascular hyperreactivity

Hypersensitivity of sensory nerves to external stimuli may contribute to neurovascular dysregulation observed in rosacea and hypertension. TRP channels are key molecular sensors for thermal, mechanical, and chemical stimuli, and their dysregulation is involved in neurogenic inflammation (30). In rosacea, human lesional skin studies have demonstrated subtype-related differences in TRP channel expression across ETR, PPR, and PHR, suggesting differential involvement of TRPV channels among clinical subtypes (38). In contrast, evidence regarding TRPA1 expression in rosacea is limited and primarily derived from experimental models, while its subtype-specific expression in human skin remains insufficiently characterized (32). In the cardiovascular system, TRP channels are involved in vascular tone regulation and neurovascular signaling. Experimental and preclinical studies indicate that TRPV1 is involved in vascular and autonomic regulation under conditions of oxidative stress or inflammation. TRPV1 dysregulation has been associated with alterations in sympathetic activity and endothelial function; however, its precise role in hypertension remains controversial and incompletely understood (39). TRPV4 channels regulate vasomotor function by modulating Ca^2+^ signaling in vascular smooth muscle cells (VSMCs) and endothelial cells (ECs) (40). Under hypertensive conditions, TRPV4 signaling may be altered, and such dysregulation may be associated with impaired vascular function. However, direct causal evidence linking TRPV4 alterations to hypertension development remains limited (22).

Activation of TRP channels on cutaneous sensory nerve endings by external factors (such as heat, emotional stress, or capsaicin) may induce the release of neuropeptides including substance P, CGRP, vasoactive intestinal peptide (VIP), and pituitary adenylate cyclase-activating polypeptide (PACAP) (41). Locally in the skin, substance P induces vasodilation via nitric oxide (NO) release from endothelial cells (ECs) (32), whereas excessive release of CGRP may contribute to increased microvascular permeability, vasodilation, tissue edema, increased blood flow, and recruitment of inflammatory cells (42). Under conditions of endothelial dysfunction, substance P acts directly on neurokinin-1 receptors (NK1R) on VSMCs, inducing Ca^2+^ influx and potentially contributing to vasoconstriction (43). Substance P can stimulate cardiac inflammatory cells to produce Ang II, potentially shifting vascular responses toward vasoconstrictive and pro-inflammatory states (44). Although CGRP plays a protective role in the circulatory system and is markedly elevated due to compensatory mechanisms (45), studies speculate that the vasodilatory protective effect may be attenuated under inflammatory remodeling and endothelial dysfunction associated with TRPV1 signaling (46). Furthermore, potent vasodilatory peptides such as PACAP and VIP exacerbate facial flushing in rosacea (32), whereas in the systemic circulation, PACAP may activate the sympathetic nervous system, potentially contributing to increased heart rate and elevated blood pressure (47). Collectively, current evidence suggests that sensory nerve hypersensitivity and TRP channel dysfunction are involved in both local neurogenic inflammation and systemic vascular dysregulation.

### 5.2 Interactions among sensory nerves, mast cells, and endothelial inflammation

In rosacea pathogenesis, the TLR2-KLK5-LL-37 axis represents a local biochemical cascade that may contribute to a pro-inflammatory microenvironment linked to neurogenic inflammation. External environmental stimuli (e.g., ultraviolet radiation, microbial stress) may activate TLR2 on keratinocytes, leading to upregulation of kallikrein-5 (KLK5). KLK5 subsequently cleaves antimicrobial peptide precursors, generating the active peptide LL-37 (48). LL-37 generated under these conditions binds to the Mas-related G-protein-coupled receptor-X2 (MRGPRX2) on mast cells, potentially enhancing their responsiveness to subsequent neurogenic stimuli (49). Under these conditions, neuropeptides released from sensory nerve endings (e.g., substance P and PACAP) may act not only on vascular targets but also on primed mast cells via MRGPRX2, thereby contributing to mast cell degranulation (33, 50). Furthermore, elevated IgE in the circulatory system and LL-37 induced by environmental stimuli may further enhance mast cell activation via their respective receptors, FcϵRI and MRGPRX2. Collectively, these multisource signals enhance mast cell-mediated neuroimmune and vascular inflammatory responses (51–53). Activated mast cells produce vascular endothelial growth factor (VEGF), platelet-derived growth factor (PDGF), fibroblast growth factor (FGF), and matrix metalloproteinase-9 (MMP-9), all of which participate in vascular remodeling and angiogenic responses. These processes contribute to persistent telangiectasia, edema, and microvascular alterations observed in rosacea (49). Activated mast cells also release mediators such as histamine, tryptase, and cytokines including IL-6 and TNF-α. IL-6 may promote ROS production in vascular endothelium and impair phosphorylation of endothelial nitric oxide synthase (eNOS), leading to decreased NO levels. This process may contribute to altered vascular reactivity and increased peripheral resistance (51). Furthermore, mast cells can locally influence the renin-angiotensin system through release of renin and chymase, potentially contributing to local Ang II generation and vasoconstrictive and profibrotic responses (54). Taken together, interactions among sensory nerves, mast cells, and endothelial cells may contribute to vascular inflammatory responses in rosacea and vascular dysfunction implicated in hypertension. However, the precise contribution of these neuroimmune interactions to disease comorbidity remains to be further elucidated.

### 5.3 Interactions among sensory nerves, myeloid immunity, and oxidative stress

Interactions among sensory nerves, myeloid immunity, and oxidative stress may be involved in inflammatory and vascular alterations in rosacea and hypertension. Current evidence suggests that neurogenic inflammation recruits and activates myeloid cells, particularly neutrophils and macrophages, which may contribute to oxidative stress, hypoxic signaling, and persistent inflammatory responses associated with vascular remodeling. Neuropeptides released by sensory nerves may attract neutrophils and macrophages into lesional skin or vascular wall areas via chemotaxis (55). In rosacea lesions, marked neutrophil infiltration and CXCL8 upregulation have been observed (56). Activated neutrophils generate ROS and release myeloperoxidase (MPO), promoting oxidative stress and excessive formation of neutrophil extracellular traps (NETs) (57, 58). In the circulatory system, MPO-derived oxidants may reduce NO bioavailability. NETs have also been implicated in endothelial dysfunction and elevated blood pressure in hypertension (59, 60). Thus, neutrophil-driven oxidative stress may exhibit context-dependent effects: in the cutaneous microenvironment, it is associated with vasodilation and dermal structural alterations, whereas in the systemic vasculature, it contributes to endothelial dysfunction, vascular fibrosis, and increased peripheral resistance.

In rosacea skin and hypertensive vascular environments, macrophages tend to exhibit a pro-inflammatory M1-skewed phenotype. Substance P from sensory nerves, acting via NK1R, may synergize with angiotensin II signaling to activate NF-κB and MAPK signaling pathways, and promote NLRP3 inflammasome assembly, leading to increased secretion of IL-1β, IL-6, and TNF-α (61). These factors may amplify neurogenic inflammation in the skin (contributing to flushing), while in the systemic vasculature they promote endothelial dysfunction and sustained eNOS inhibition, potentially contributing to hypertension pathophysiology (51). Moreover, macrophage activation is often linked to hypoxic microenvironments. Hypoxia may induce HIF-1α activation, driving metabolic reprogramming (62). This process may contribute to increased VEGF expression in rosacea, leading to pathological angiogenesis (63), whereas in hypertension it may be associated with oxidative endothelial injury (64). Additionally, M1-polarized macrophages release MMP-9, contributing to extracellular matrix remodeling. In rosacea, MMP-9 may degrade dermal collagen and elastin, reducing perivascular support and promoting persistent erythema (56). In hypertension, macrophage-derived MMP-9 may contribute to vascular stiffness (65). Collectively, myeloid cell-driven oxidative and inflammatory pathways may reflect overlapping pathophysiological processes in rosacea and hypertension, although current evidence remains largely indirect and does not establish a causal mechanistic link between cutaneous inflammation and blood pressure regulation.

### 5.4 Adaptive immune responses and vascular inflammation under chronic neural stress

Chronic neural stress may be associated with adaptive immune responses involving both T cells and B cells, which contribute to inflammatory processes observed in rosacea and hypertension. Sensory nerve endings in a persistently hyperresponsive state continuously release neuropeptides, which together with norepinephrine released by sympathetic nerves may modulate dendritic cell (DC) function and influence pro-inflammatory T-cell differentiation (66). Meanwhile, excessive ROS generated during neuroimmune inflammatory responses may induce oxidative modification of endogenous proteins, which can be captured and presented by DCs as neoantigens (67). Similar immune-inflammatory processes may occur in both chronic cutaneous inflammation in rosacea and vascular injury in hypertension, potentially linking innate immune activation with adaptive immune responses. On this basis, the Th17/IL-17 signaling pathway may participate in inflammatory and vascular responses shared by rosacea and hypertension. Activated DCs may promote T-cell polarization toward the Th17 subset, and IL-17 released from Th17 cells exerts pro-inflammatory effects across different tissue environments: in rosacea, it may contribute to persistent erythema through neutrophil and macrophage recruitment (68); in hypertension, IL-17 may act on vascular smooth muscle cells, potentially contributing to vascular proliferation and increased peripheral resistance, and may also participate in salt-sensitive blood pressure regulation through effects on renal tubular sodium transporters (69, 70). This pleiotropic immune response may partly explain the observed association between rosacea severity and systemic vascular inflammation.

**Figure 2:**
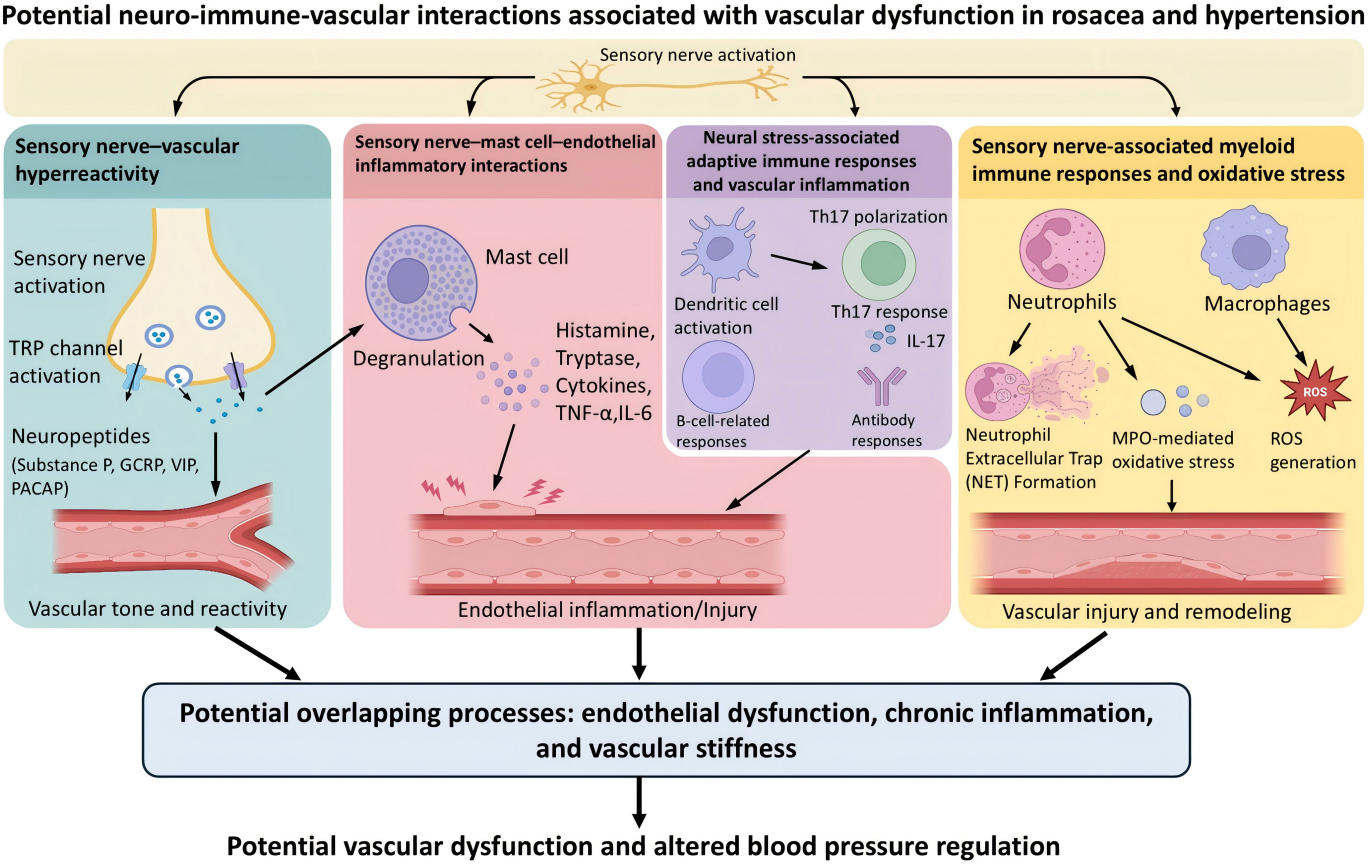
Potential neuro-immune-vascular interactions associated with vascular dysfunction in rosacea and hypertension. This figure summarizes putative neuro-immune-vascular processes linking rosacea and hypertension. Sensory nerve activation and TRP channel dysregulation are associated with neuropeptide release, mast cell activation, myeloid immune responses, oxidative stress, and adaptive immune alterations. These processes converge on endothelial dysfunction and chronic inflammation, which may contribute to vascular stiffness and altered blood pressure regulation. The proposed interactions are hypothesis-driven and do not imply a unified causal pathway.

Another potential immune-regulatory component involves B-cell activation. Although B-cell markers such as CD20 are increased in rosacea lesions, suggesting local infiltration, the functional relevance and overall role of B cells remain poorly defined (71). In other inflammatory diseases, TLR-mediated signaling has been shown to influence B-cell activation and antibody responses, including the generation of autoantibodies under conditions of chronic immune stimulation, although this has not been established in rosacea. Whether rosacea-associated antibodies target self-antigens or Demodex antigens, and whether such targeting engages TLR pathways to promote tissue remodeling, remain to be directly validated by experimental studies (72). Furthermore, regulatory B cells (Bregs) exert anti-inflammatory effects primarily through IL-10 production, thereby modulating Th1- and Th17-mediated immune responses. Conversely, excessive Th1/Th17-mediated inflammatory responses may impair the differentiation and function of Bregs, leading to reduced IL-10 production. This imbalance may further weaken anti-inflammatory regulation and potentially contribute to persistent vascular dysfunction and remodeling (73, 74). Collectively, adaptive immune alterations associated with chronic neuroimmune stimulation may contribute to persistent inflammatory responses in rosacea, while current evidence supporting a direct role in hypertension or disease comorbidity remains limited and requires further validation. These interconnected neuro-immune-vascular processes and their potential convergence on vascular dysfunction are summarized in **Figure 2**.

## 6 Potential intervention strategies based on shared mechanisms

As discussed above, rosacea and hypertension share significant common mechanisms in terms of neurovascular dysregulation, cellular immunity, and inflammatory responses. These shared pathophysiological mechanisms offer new perspectives for re-evaluating and optimizing clinical treatment strategies. The following section will explore the therapeutic implications arising from these shared mechanisms.

### 6.1 Neurovascular-targeted therapy

Studies have shown that emotional fluctuations or external environmental stimuli can induce sympathetic nervous system excitation. This excited state mediates cutaneous vasodilation via activation of β_2_-adrenergic receptors and simultaneously amplifies the neuropeptide-driven neurovascular coupling response. This cascade of neurogenic inflammation and dysregulation of vasomotor balance collectively exacerbates the paroxysmal flushing and persistent erythema characteristic of rosacea (75). A systematic review indicated that non-selective or partially selective β-blockers (e.g., propranolol, nadolol, carvedilol) can be used to improve rosacea-associated flushing and erythema, with notable short-term efficacy (76). Among these, carvedilol significantly suppressed the expression of TLR2, KLK5, and LL-37 in a rosacea-like inflammatory model, thereby alleviating the inflammatory response (77). Therefore, in patients with comorbid hypertension and rosacea, these agents may exert dual regulatory effects; however, their use should be carefully individualized based on cardiovascular and pulmonary status. In particular, non-selective β-blockers may be contraindicated in patients with asthma, bradycardia, or hypotension, and require cautious use in clinical practice. Botulinum toxin, a neuromodulator, has demonstrated efficacy in the treatment of rosacea. Its mechanism primarily involves blocking acetylcholine receptors and downregulating the release of neuropeptides such as substance P and CGRP, thereby alleviating neurogenic inflammation and abnormal vasodilation (78). Clinical studies have confirmed that intradermal injection of botulinum toxin type A can effectively improve paroxysmal flushing, persistent erythema, and burning sensation in patients, with the therapeutic effect lasting for several months (14). The CGRP signaling pathway may exert context-dependent effects in rosacea and hypertension. While CGRP antagonists have shown efficacy in migraine and may alleviate vasodilation-related symptoms in rosacea, CGRP also plays a protective role in vascular homeostasis. Therefore, inhibition of CGRP signaling may carry potential cardiovascular risks. This suggests that when CGRP antagonists are used for skin-related diseases, careful monitoring of blood pressure and systemic risks is warranted (79). Similarly, NK1R antagonists may alleviate neurogenic inflammation by blocking substance P signaling. They have shown potential in conditions such as prurigo nodularis and psoriasis-associated pruritus, and may offer new insights for the regulation of vascular inflammation in both rosacea and hypertension in the future (80, 81). Furthermore, activation of TRP channels (e.g., TRPV1) promotes the release of vasoactive neuropeptides such as CGRP and substance P; inhibiting their activation may reduce neuropeptide-mediated vasodilation and inflammatory responses, making TRPV1 antagonists a potential therapeutic target for rosacea (36, 82). Although some studies suggest that TRPV1 antagonists do not affect resting blood pressure, these channels are involved in baroreceptor reflexes and may influence the sensing and regulation of blood pressure fluctuations (83). Therefore, further elucidation of the specific mechanisms of TRP channels in the pathogenesis of rosacea and hypertension will help advance precision therapeutic strategies targeting such comorbid mechanisms.

### 6.2 Targeting immune-inflammatory pathways

#### 6.2.1 TLR2-KLK5-LL-37 axis

Targeting the TLR2–KLK5–LL-37 axis represents a well-established therapeutic strategy in rosacea. Currently available agents, including azelaic acid, ivermectin, and tetracyclines, have been shown to modulate this pathway to varying degrees (84). In addition, colchicine has been reported to ameliorate rosacea-like inflammation by inhibiting TLR2-mediated neutrophil activation and NET formation, thereby attenuating the TLR2–LL-37 inflammatory feedback loop (85). In the cardiovascular system, LL-37 has been suggested in experimental and preclinical studies to exert pro-inflammatory effects, including the promotion of platelet activation and endothelial dysfunction (86). These observations indicate a potential involvement of LL-37 in vascular inflammatory processes; however, evidence linking this pathway to hypertension remains indirect and insufficient to support a definitive mechanistic role. Therefore, while the TLR2–KLK5–LL-37 axis is a well-established pathogenic pathway in rosacea, its relevance to hypertension is currently speculative and should be interpreted as a hypothesis-generating extension rather than a confirmed shared mechanism.

#### 6.2.2 IL-17 pathway

IL-17 is a central pro-inflammatory cytokine implicated in both rosacea and hypertension. IL-17 inhibitors have been well established in Th17-related diseases such as psoriasis (87), and case reports suggest they are also effective in refractory rosacea (88). Furthermore, a clinical trial involving 24 patients with papulopustular rosacea demonstrated that secukinumab (300 mg weekly for 5 weeks, followed by monthly maintenance) significantly improved papule symptoms and disease severity (89). In hypertension research, IL-17 deficiency has been shown to exert blood pressure-lowering effects in animal models (90). However, current evidence is largely derived from case reports and animal studies, and the causal relationship of this pathway in the comorbidity requires further validation through clinical cohort studies.

#### 6.2.3 NLRP3/IL-1β pathway

The NLRP3/IL-1β pathway is involved in inflammatory regulation in both diseases. Studies have shown that MCC950, an NLRP3 inhibitor, lowers blood pressure and suppresses vascular inflammation in hypertensive models (91). Canakinumab, an IL-1β monoclonal antibody, significantly reduces the risk of cardiovascular events without altering lipid profiles, indicating that specific inhibition of IL-1β exerts protective effects independent of conventional antihypertensive agents (92). Similarly, in rosacea models, licochalcone A inhibits NLRP3 activation, reduces IL-1β release, and ameliorates rosacea-associated erythema and telangiectasia (93). These findings suggest that NLRP3 inflammasome inhibitors hold potential therapeutic value in the management of both rosacea and hypertension.

#### 6.2.4 JAK/STAT pathway

The JAK/STAT pathway plays a critical role in the pathogenesis and therapeutic research of both rosacea and hypertension. In rosacea, a retrospective study of 21 patients treated with tofacitinib showed that oral JAK inhibitors improved erythema and papule symptoms within weeks (94). Furthermore, network pharmacology analysis revealed that POSTN is highly expressed in rosacea and exacerbates inflammatory responses, promotes immune cell infiltration, and enhances angiogenesis by activating the JAK2/STAT3 pathway, whereas silencing the POSTN gene alleviated the LL-37-induced rosacea-like phenotype (95). In the field of hypertension, a study based on a metabolic hypertension rat model demonstrated that the deubiquitinating enzyme USP18 curbs hypertension progression by negatively regulating the JAK/STAT pathway (96). Clinical observations have found that statins may improve vascular endothelial dysfunction in elderly patients with essential hypertension via this pathway (97). Meanwhile, certain herbal formulas and their active components (e.g., tanshinone, astragaloside IV) have also been shown to regulate the JAK2/STAT3 pathway, suggesting potential therapeutic value for targeting this pathway with traditional Chinese medicine and phytochemicals in the future (98). In summary, the JAK/STAT pathway serves as an important molecular bridge connecting the inflammatory and vascular abnormality mechanisms of the two diseases, and inhibitors targeting this pathway offer new research directions for interventions in rosacea and hypertension.

### 6.3 Lifestyle intervention

Lifestyle intervention may exert beneficial effects on both rosacea and hypertension by modulating multiple shared pathophysiological pathways, including systemic inflammation, oxidative stress, sympathetic overactivity, and endothelial dysfunction. Studies have shown that adherence to an anti-inflammatory dietary pattern, characterized by high intake of vegetables, fruits, whole grains, and omega-3 fatty acids, reduces systemic inflammatory markers and improves endothelial function. These changes are of significant relevance both for rosacea, which is centrally characterized by chronic inflammation and enhanced vascular reactivity, and for hypertension, where inflammation contributes to the development of arterial stiffness (99–101). Research indicates that omega-3 fatty acids improve vascular endothelial function and reduce vascular inflammation by inhibiting the production of pro-inflammatory cytokines (e.g., IL-6, TNF-α), lowering oxidative stress levels, and increasing NO bioavailability (102). Furthermore, some studies suggest that omega-3 fatty acids may also alleviate cutaneous inflammation and improve clinical manifestations of certain inflammatory skin diseases by modulating inflammatory signaling pathways (e.g., NF-κB) and lipid-mediated immune responses (103). Although direct interventional studies specifically targeting rosacea remain relatively limited, these findings suggest that anti-inflammatory diets and omega-3 fatty acid supplementation may concurrently influence systemic vascular inflammation and cutaneous inflammatory responses, thereby potentially providing synergistic benefits for patients with comorbid rosacea and hypertension. Controlling common risk factors such as smoking, alcohol consumption, and spicy foods may offer beneficial strategies for the integrated management of rosacea and hypertension by reducing neurovascular irritation and maintaining vascular homeostasis (84, 104–106). Reducing sodium intake has been shown to effectively lower blood pressure by reducing Th17 cell activation, thereby decreasing the production and release of IL-17A, significantly lowering blood pressure and reducing vascular inflammation (107, 108). Given that the pathogenesis of rosacea involves chronic skin inflammation and pro-inflammatory cytokines, reducing sodium intake may theoretically exert an adjunctive ameliorating effect on rosacea symptoms by decreasing systemic and local pro-inflammatory stimuli, as well as reducing fluid retention and vascular reactivity. Furthermore, psychological stress is a common trigger that exacerbates both hypertension and rosacea through activation of the HPA axis and the sympathetic nervous system. Therefore, stress management strategies such as mindfulness, yoga, and cognitive-behavioral therapy may help restore autonomic nervous system balance, rendering them potentially innovative non-pharmacological approaches for the management of this comorbidity (109, 110).

## 7 Conclusion and perspectives

Rosacea and hypertension have traditionally been considered distinct disorders affecting different organ systems; however, emerging evidence suggests that they may share partially overlapping neuro-immune-vascular regulatory features, including sensory nerve activation, neuropeptide signaling, immune-inflammatory responses, oxidative stress, endothelial dysfunction, and vascular remodeling. These observations collectively support a conceptual framework in which cutaneous and systemic vascular alterations may reflect interconnected, context-dependent biological processes rather than isolated disease mechanisms. Given the heterogeneity of evidence across epidemiological, cutaneous, and cardiovascular domains, we have constructed a summary table (**Supplementary Table 1**) that delineates the evidence source, model system, and strength of evidence for each proposed neuro-immune-vascular mechanism, as well as the key limitations of the current data.

Current evidence suggests that neuropeptide-mediated neuroimmune activation may contribute to cutaneous inflammation and vascular dysregulation in rosacea, and may also be associated with systemic low-grade inflammation and altered vascular reactivity observed in hypertension. Notably, neuropeptides such as CGRP may exert context-dependent effects across tissue environments, with pro-inflammatory or vasodilatory actions in the skin and compensatory vascular effects in the systemic circulation. These properties highlight the complexity of interpreting shared neurovascular features across disease states. In addition, converging inflammatory pathways involving mast cells, myeloid immune responses, oxidative stress, and adaptive immune activation may participate in sustained vascular inflammation and remodeling in both conditions. Lifestyle factors and neuroimmune modulation may represent potential complementary considerations for disease management, although these implications remain exploratory and require further validation in future studies.

Nevertheless, current evidence is primarily derived from epidemiological associations, experimental models, and indirect mechanistic studies. Direct evidence establishing a causal relationship between rosacea and hypertension is lacking. Accordingly, the present interpretation should be regarded as hypothesis-driven rather than confirmatory of a unified causal pathway. Future longitudinal and mechanistic studies are needed to clarify the temporal and biological relationships between these conditions and to determine whether targeting shared neuro-immune-vascular pathways has clinical relevance.

## Supplementary material

Supplementary Table 1 Summary of evidence sources and strength supporting each proposed mechanism.
